# Stabilizing Constructs through Collaboration across Different Research Fields as a Way to Foster the Integrative Approach of the Research Domain Criteria (RDoC) Project

**DOI:** 10.3389/fnhum.2016.00309

**Published:** 2016-06-28

**Authors:** Jacqueline A. Sullivan

**Affiliations:** Department of Philosophy, Rotman Institute of Philosophy, University of Western OntarioLondon, ON, Canada

**Keywords:** construct, DSM, experimental paradigm, integration, RDoC, validity

## Introduction

More than 450 million people worldwide suffer from neuropsychiatric disorders and the numbers continue to rise (WHO, 2016). In 2010, aiming to solve the global mental health crisis and advance psychiatry toward a *precision medicine* approach, the US National Institute for Mental Health (NIMH) initiated the *Research Domain Criteria* (RDoC) *Project* (Cuthbert and Insel, 2013). Scientists at NIMH importantly recognize that understanding and explaining psychopathological phenomena requires input from different areas of science that investigate the role of different units of analysis (e.g., genes, cells, systems) in the production of organism-level behavioral functions. The RDoC matrix is put forward as a context for integrating results from these different sciences into a taxonomy of putatively *valid* constructs they purportedly share in common. It is intended to facilitate the development of “integrative psychobiological explanations” of those behavioral functions designated by the constructs (Cuthbert and Kozak, 2013, p. 931; See also Sanislow et al., 2010). Such explanations, by shedding light on the mechanisms of these functions, will enable investigators to pinpoint viable targets for therapeutic intervention in cases in which these functions are disrupted.

The RDoC project is still in its infancy and its proponents recognize that it has much room for improvement (Casey et al., 2014). To date, it has been criticized for being “braincentric” and decontextualizing mental disorders from their bodily, social, and environmental contexts (e.g., Whooley, 2014; Bernard and Mittal, 2015). Although proponents of RDoC claim that one of its crucial aims is to integrate various areas of science, an obstacle to this integration is the lack of *construct stability* in psychology and neuroscience. In this article, I explain why stabilizing constructs is important to the success of the RDoC initiative and identify one measure for facilitating such stability.

## The RDoC matrix

The RDoC Matrix consists of a table in which findings from psychology and neuroscience may be organized. Five broad domains of behavioral functioning are identified in the rows of the first column of the matrix: (1) *positive*, (2) *negative valence systems*, (3) *cognitive*, (4) *social processing*, and (5) *arousal/modulatory systems*. A selection of constructs designating *some* behavioral functions currently under study across psychology and neuroscience are identified and classified under one of each of the 5 domains. For example, *attention, perception, declarative memory, language, cognitive control*, and *working memory* are classified as cognitive systems. Negative valence systems include the constructs of *acute threat, potential threat, sustained threat, loss*, and *frustrative nonreward*. Each construct is also given a general definition. In most cases, these definitions make reference to neural, psychological, and behavioral processes associated with the construct. For example, fear is characterized as involving “activation of the brain's defensive motivational system to promote” protective behaviors (i.e., neural processes), “a pattern of adaptive responses to conditioned and unconditioned threat stimuli” (i.e., behavioral/psychological processes) and possibly “internal representations and cognitive processing” (i.e., psychological processes) (http://www.nimh.nih.gov/research-priorities/rdoc/constructs/acute-threat-fear.shtml).

The columns of the matrix reflect the fact that research on domains of behavioral functioning spans multiple levels of organization from genes to cells to networks to behavior and includes multiple different areas of science (e.g., psychology, systems neuroscience, and neurobiology). An additional column labeled “paradigms” is where *experimental paradigms*, standard procedures for producing, detecting and measuring behavioral functions that correspond to the constructs in the matrix, are placed. The Stroop Task is an experimental paradigm historically used to investigate selective attention in human subjects. Fear-conditioning paradigms, in contrast, are used to study fear in humans and non-human mammals.

How will RDoC facilitate progress in understanding disturbances in behavioral functioning? Investigators working in those sciences represented in the matrix use experimental paradigms to produce, detect and measure instances of behavioral functions that correspond to RDoC constructs. The constructs are essentially labels that are linked up with (1) experimental paradigms used to investigate the behavioral functions designated by those labels and (2) units of analysis that have been implicated in the production of behavioral functions designated by those labels. Although proponents of RDoC remain vague about the details, at some point research findings inputted into the matrix are supposed to result in integrative psychobiological or mechanistic explanations that describe the physical components and processes that bring about the functions designated by RDoC constructs (Cuthbert and Kozak, 2013). Knowledge about mechanisms is in turn supposed to foster the development of therapeutic interventions in cases in which behavioral functions are disrupted, with the aim of restoring normal functioning in those individuals.

## Obstacles to integration

We should not expect *integration* to be an emergent feature of the juxtaposition of a mass of research findings emanating from psychology and neuroscience. The explanatory and conceptual integration that RDoC is supposed to deliver instead requires intensive collaborative efforts on the part of investigators working in areas of science that investigate RDoC constructs. To understand why, a closer look at explanatory, conceptual and investigative practices in psychology and neuroscience is relevant.

Explanations in psychology have been characterized as *explanations by functional analysis* (Fodor, 1968; Cummins, 1983). These explanations involve ascriptions of functions to organisms and the abstract identification of the sub-capacities that bring these functions about without regard for anatomical, structural, biochemical, or physiological facts about the brain and nervous system. They often consist of box-and-arrow diagrams where boxes stand in for *psychological* capacities and arrows represent information flow through the system from stimulus inputs to behavioral outputs. An explanation of *attention* by functional analysis, for example, may describe a “short term storage” system that receives information from the periphery and sends it through a “selective filter” that determines what information is received by systems downstream (Broadbent, 1958). Explanations in neuroscience, in contrast, are described as *mechanistic* insofar as they identify the physical parts (e.g., systems, cells, molecules) and processes (e.g., activation, firing, phosphorylation) that realize organism-level functions (Craver, 2007; Bechtel, 2008). Part of a mechanistic explanation of *attention*, for example, may describe activation of dopamine receptors and depolarization of medium spiny neurons in the nucleus accumbens of the basal forebrain.

A prerequisite for integrating explanations by functional analysis with mechanistic explanations is “connectability” (Nagel, 1961). More specifically, the terms designating cognitive capacities in an explanation by functional analysis must have roughly the same referents as the terms designating cognitive capacities in a mechanistic explanation. To refer back to the previous example, an explanation by functional analysis that contains the term *attention* ought to refer to the same phenomenon as a mechanistic explanation that contains that term. Terms designating cognitive capacities are the common denominator between the two forms of explanation and satisfying the connectability condition requires that the terms designate the same thing. Otherwise, rather than explanatory integration, terms in one area of science are eliminated and replaced with those of another.

The RDoC task forces implicitly realize that satisfaction of the connectability condition is required for explanatory integration, as is evidenced by the fact that they have sought to deploy strategies to *stabilize* RDoC constructs, while simultaneously acknowledging that the constructs are heuristics that may warrant revision in light of future discovery (Cuthbert and Insel, 2013). However, stabilizing constructs requires more than a small group of investigators selecting a set of terms that are used across different areas of science and providing them with definitions broad enough to accommodate different uses of these terms. As RDoC's creators acknowledge, it is not a project that will prove ultimately successful if it isolates itself from those very sciences poised to shed light on the kinds of questions it is designed to answer (e.g., Simmons and Quinn, 2014). RDoC's success is instead contingent on a large-scale revolution in the mind-brain sciences to collectively stabilize constructs so that conceptual and explanatory integration are possible. One component of this revolution has to be coordination across investigators working *in the same* and *different* areas of science to come to specific agreement about (1) how to define terms designating behavioral functions, (2) what the best experimental paradigms for studying a given behavioral function are, and (3) when two experimental paradigms may be said to produce, detect and measure roughly the same function.

Do we encounter such coordination within or across psychology and neuroscience currently? Let's begin by considering cognitive psychology. One of its paradigmatic features is the importance placed on engaging in rigorous task analyses to determine the component cognitive processes operative when subjects are trained and tested in experimental paradigms. This ought to mean that intra-lab strategies are in place to ensure that experimental paradigms measure the functions they are intended to measure, which ought to contribute positively to construct stabilization. While this is true, inter-lab practices are not necessarily conducive to stability. For example, two investigators may be interested in investigating the same function, but disagree about the most suitable task for this purpose. Since stimuli and task demands may differ radically between tasks, it is difficult to determine if the same component cognitive processes are involved in each task and whether they measure the same thing (Sullivan, 2009; Lilienfeld, 2014; Lilienfeld and Treadway, 2016). Investigators often disagree about which component cognitive processes are involved in a task and the behavioral data often are compatible with multiple different explanations by functional analysis.

Although we may be optimistic that cognitive neuroscience will provide the fMRI or other brain data requisite for resolving such problems of underdetermination, it has its own troubles with respect to construct stabilization. Many cognitive neuroscientists do not aim to identify the component cognitive processes thought to be engaged in experimental tasks nor to determine how the variables manipulated in an experiment affect these processes (Sullivan, 2014a,b). In fact, when evaluating or comparing tasks it is more common for investigators to rely on “intuitive judgments” about the processes involved rather than look to “formal theories from cognitive psychology” for guidance (Poldrack, 2010, p. 149).

We also find rampant methodological pluralism in those areas of cellular and molecular neuroscience that investigate cognition and behavior. Individual researchers vary experimental paradigms and protocols used to produce, measure and detect behavioral functions. Yet even subtle differences in experimental protocols can impact the mechanisms productive of those functions, prompting uncertainty as to whether different laboratories are investigating the same phenomenon. In some cases, investigators may be unclear what function they are discovering the mechanisms of (Sullivan, 2010), but given that they do not regard understanding component cognitive processes as relevant to their mechanistic explanatory goals, they see no need to look toward psychology for guidance.

## Conclusion

If construct instability across psychology and neuroscience is as pervasive as these facts about practice suggest, then stabilizing them for the purposes of conceptual and explanatory integration is going to require scientists engaged in research relevant to investigating the domains of functioning identified in the matrix—including research on domains currently and problematically absent from the matrix, like *motor functions* (Bernard and Mittal, 2015)—to interact with each other in the trenches to do hard work (Bilder et al., 2013). To date, RDoC has pointed to large-scale meta-analyses as a primary way forward. However, insofar as meta-analyses abstract away from conceptual and experimental practices operative within and across different areas and laboratories in psychology and neuroscience, they will not yield *valid* constructs. Details about features of individual experiments matter for comparing data across laboratories and determining if the same capacities are under study and the same mechanisms are operative. While amassing discordant evidence under a set of common labels may result in testable hypotheses it will not directly shed light on real divisions in the causal structure of the world.

**Figure 1:**
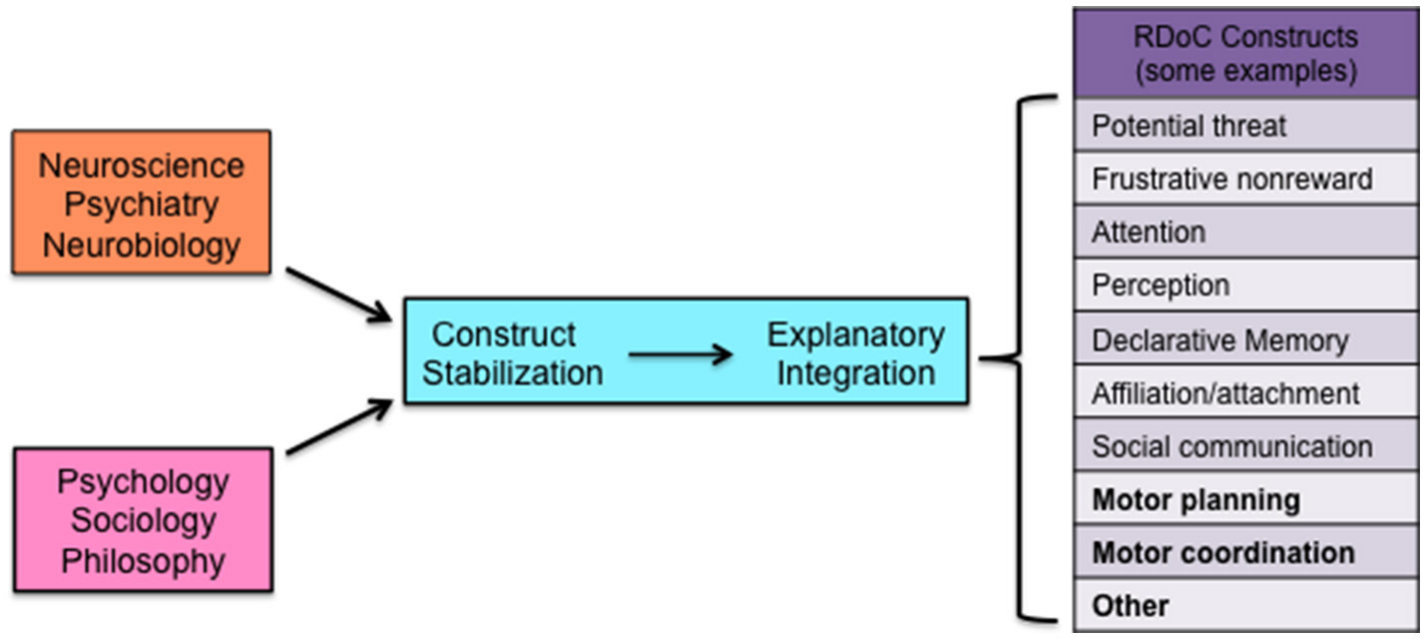
**Schematic representation of different areas of science contributing collectively to construct stabilization, which facilitates explanatory integration and the development of integrated valid constructs**. Some examples of current RDoC constructs are identified in the figure in plain typeface. Constructs currently not represented in the RDoC matrix and others to be added are identified in boldface.

RDoC's success requires instead deliberate efforts across the relevant sciences and humanities to collectively stabilize its constructs (Figure 1). One plausible way forward is to create networks of investigators representing a diverse array of perspectives on behavioral and psychological functions and regularly bring them together to facilitate discussions about what the relevant constructs are, how to investigate them, how to stabilize them, and related issues (See for example http://www.rotman.uwo.ca/events-2/rethinking-the-taxonomy-of-psychology-conference/). This is the RDoC model on a larger scale and with greater inclusivity, but only by means of broader collaborative efforts may we hope to ensure the realization of RDoC's positive aims.

## Author contributions

The author confirms being the sole contributor of this work and approved it for publication.

### Conflict of interest statement

The author declares that the research was conducted in the absence of any commercial or financial relationships that could be construed as a potential conflict of interest.

## References

[B1] Bechtel W. (2008). Mental Mechanisms: Philosophical Perspectives on Cognitive Neuroscience. New York, NY: Taylor and Francis.

[B2] Bernard J. A. Mittal V. A. (2015). Updating the research domain criteria: the utility of a motor dimension. Psychol. Med. 45, 2685–2689. 10.1017/S0033291715000872 26005109 PMC4565742

[B3] Bilder R. Howe A. Sabb F. (2013). Multilevel models from biology to psychology: mission impossible? J. Abnorm. Psychol. 122, 917–927. 10.1037/a0032263 23647123

[B4] Broadbent D. (1958). Perception and Communication. London: Pergamon Press.

[B5] Casey B. J. Oliveri M. E. Insel T. (2014). A neurodevelopmental perspective on the research domain criteria (RDoC) framework. Biol. Psychiatry 76, 350–353. 10.1016/j.biopsych.2014.01.006 25103538

[B6] Craver C. (2007). Explaining the Brain: Mechanisms and the Mosaic Unity of Neuroscience. Oxford, UK: Oxford University Press.

[B7] Cummins R. (1983). The Nature of Psychological Explanation. Cambridge, MA: MIT Press.

[B8] Cuthbert B. Insel T. (2013). Toward the future of psychiatric diagnosis: the seven pillars of RDoC. BMC Med. 11:126. 10.1186/1741-7015-11-126 23672542 PMC3653747

[B9] Cuthbert B. Kozak M. (2013). Constructing constructs for psychopathology: the NIMH research domain criteria. J. Abnorm. Psychol. 122, 928–937. 10.1037/a0034028 24016027

[B10] Fodor J. (1968). Psychological Explanation: An Introduction to the Philosophy of Psychology. New York, NY: Random House.

[B11] Lilienfeld S. (2014). The Research Domain Criteria (RDoC): an analysis of methodological and conceptual challenges. Behav. Res. Ther. 62, 129–139. 10.1016/j.brat.2014.07.019 25156396

[B12] Lilienfeld S. Treadway M. (2016). Clashing Diagnostic Approaches: DSM-ICD Versus RDoC. Annu. Rev. Clin. Psychol. 12, 435–463. 10.1146/annurev-clinpsy-021815-093122 26845519 PMC5154554

[B13] Nagel E. (1961). The Structure of Science: Problems in the Logic of Scientific Explanation. New York, NY: Harcourt, Brace & World.

[B14] Poldrack R. (2010). Subtraction and Beyond: The Logic of Experimental Designs for Neuroimaging, in Foundational Issues in Human Brain Mapping, eds Hanson S. Bunzl M. (Cambridge: MIT Press), 147–159.

[B15] Sanislow C. A. Pine D. S. Quinn K. Kozak M. Garvey M. Heinssen R.. (2010). Developing constructs for psychopathology research: research domain criteria. J. Abnorm. Psychol. 119, 631–639. 10.1037/a0020909 20939653

[B16] Simmons J. Quinn K. (2014). The NIMH Research Domain Criteria (RDoC) Project: implications for genetics research. Mamm. Genome 25, 23–31. 10.1007/s00335-013-9476-9 24085332

[B17] Sullivan J. (2009). The multiplicity of experimental protocols: a challenge to reductionist and nonreductionist models of the unity of science. Synthese 167, 511–539. 10.1007/s11229-008-9389-4

[B18] Sullivan J. (2010). Reconsidering spatial memory and the morris water maze. Synthese 177, 261–283. 10.1007/s11229-010-9849-5

[B19] Sullivan J. (2014a). Is the next frontier in neuroscience a decade of the mind, in Brain Theory, ed Wolfe C. (New York, NY: Palgrave-MacMillan), 45–67.

[B20] Sullivan J. (2014b). Stabilizing mental disorders: prospects and problems, in Classifying Psychopathology: Mental Kinds and Natural Kinds, eds Kincaid H. Sullivan J. (Boston, MA: MIT Press), 257–281.

[B22] WHO (2016). Mental Disorders [Fact Sheet]. Available online at: http://www.who.int/mediacentre/factsheets/fs396/en/

[B21] Whooley O. (2014). Nosological reflections: the failure of DSM-5, the emergence of RDoC, and the decontextualization of mental distress. Soc. Ment. Health 4, 92–110. 10.1177/2156869313519114

